# A Distinct Esophageal mRNA Pattern Identifies Eosinophilic Esophagitis Patients With Food Impactions

**DOI:** 10.3389/fimmu.2018.02059

**Published:** 2018-11-05

**Authors:** Benjamin F. Sallis, Utkucan Acar, Kelsey Hawthorne, Stephen J. Babcock, Cynthia Kanagaratham, Jeffrey D. Goldsmith, Rachel Rosen, Jon A. Vanderhoof, Samuel Nurko, Edda Fiebiger

**Affiliations:** ^1^Division of Gastroenterology, Hepatology and Nutrition, Boston Children's Hospital, Boston, MA, United States; ^2^Center for Motility and Functional Gastrointestinal Disorders Boston Children's Hospital, Boston, MA, United States; ^3^Aerodigestive Center, Boston Children's Hospital, Boston, MA, United States; ^4^Department of Pathology, Boston Children's Hospital, Boston, MA, United States

**Keywords:** eosinophilic esophagitis, food impaction, machine learning classification, medical algorithm, esophageal motility, eosinophils

## Abstract

Eosinophilic esophagitis (EoE), a Th2-type allergic immune disorder characterized by an eosinophil-rich esophageal immune infiltrate, is often associated with food impaction (FI) in pediatric patients but the molecular mechanisms underlying the development of this complication are not well understood. We aim to identify molecular pathways involved in the development of FI. Due to large variations in disease presentation, our analysis was further geared to find markers capable of distinguishing EoE patients that are prone to develop food impactions and thus expand an established medical algorithm for EoE by developing a secondary analysis that allows for the identification of patients with food impactions as a distinct patient population. To this end, mRNA patterns from esophageal biopsies of pediatric EoE patients presenting with and without food impactions were compared and machine learning techniques were employed to establish a diagnostic probability score to identify patients with food impactions (EoE+FI). Our analysis showed that EoE patients with food impaction were indistinguishable from other EoE patients based on their tissue eosinophil count, serum IgE levels, or the mRNA transcriptome-based p(EoE). Irrespectively, an additional analysis loop of the medical algorithm was able to separate EoE+FI patients and a composite FI-score was established that identified such patients with a sensitivity of 93% and a specificity of 100%. The esophageal mRNA pattern of EoE+FI patients was typified by lower expression levels of mast cell markers and Th2 associated transcripts, such as *FCERIB, CPA3, CCL2, IL4*, and *IL5*. Furthermore, lower expression levels of regulators of esophageal motility (*NOS2* and *HIF1A*) were detected in EoE+FI. The EoE+FI -specific mRNA pattern indicates that impaired motility may be one underlying factor for the development of food impactions in pediatric patients. The availability of improved diagnostic tools such as a medical algorithm for EoE subpopulations will have a direct impact on clinical practice because such strategies can identify molecular inflammatory characteristics of individual EoE patients, which, in turn, will facilitate the development of individualized therapeutic approaches that target the relevant pathways affected in each patient.

## Introduction

Eosinophilic esophagitis (EoE) is an allergic disorder that is characterized by an eosinophil-rich immune infiltrate of the esophagus. Recent epidemiological reports estimate the incidence of EoE at 1/10,000 new cases per year with the current prevalence of EoE as 25.9–56.7/100,000 in the United States and suggest a rapid increase in incidence in developed countries (1–4).

EoE remains a challenge to diagnose and manage due to the high clinical variations of the disease (5–7). The diagnosis is made clinicopathologically by demonstrating at least 15 eosinophils per high-power field in at least one esophageal biopsy, along with the presence of typical EoE symptoms (8). Evaluation of 2–4 biopsies from both proximal and distal esophagus is recommended because of the patchy nature of the immune infiltrate in EoE (9). Symptomology is age and gender dependent with younger children commonly presenting with nonspecific symptoms such as failure to thrive, feeding difficulties, and choking on solid foods. Older children on the other hand, more capable of communicating their symptoms, frequently complain of abdominal pain, dysphagia, and vomiting (10–13). In contrast to children, adolescents and adults typically present with symptoms more specific to esophageal dysfunction and ongoing fibrosis, such as dysphagia, food impaction, and esophageal strictures (10, 14). In addition to the wide array of presenting symptoms the biological mechanisms underlying disease onset and progression are varied, which has led to the definition of many EoE subpopulations (15). This increasing prevalence and the heterogeneous nature of the disease underlie the pressing need to improve disease management, treatment, and diagnosis.

Currently, the use of machine learning diagnostic algorithms using mRNA pattern stamps is at the forefront of emerging diagnostic strategies for EoE. These approaches rely on an automatized evaluation of the transcriptional profile of the inflamed esophagus to calculate probability scores (16, 17). The use of a machine learning approach allows for an unbiased assessment of patient biopsies independent of human error and is designed to be self-improving with the addition of data from new patients as they become available.

The automatization of data analysis in EoE also opens the possibility of an individualized approach to EoE diagnostics and therapy. This strategy will allow clinicians to evaluate the phenotype of EoE presentation of each patient and potentially assign patients into subpopulations of EoE. Currently, multiple subpopulations of EoE patients are defined by traits such as clinical symptoms, responses to therapies (PPI-REE), and/or underlying gene expression patterns (LTC4S-EoE, IL23-EoE, iNKT-EoE, IGHEhi-EoE) (15–20). In a recently published medical algorithm, a diagnostic score for patients with increased local IgE production has been developed as a secondary diagnosis loop to identify patients that potentially suffer from local esophageal allergies (17). Further expansion of this algorithm to define additional subpopulations of EoE has the potential to improve the understanding of the disease, and to assist in the prediction of symptom onset, response to therapy, and stratification of the underlying EoE etiology.

A significant portion of patients with EoE suffer from food impactions, making EoE the leading cause of food impaction and dysphagia in the pediatric population (21). Nevertheless, the pathologic mechanisms that lead to food impactions in EoE are not well understood (22, 23). Among patients with EoE, food impaction can result from both obstructive anatomical features of the esophagus, such as esophageal stenosis, narrow-caliber esophagus, and strictures, as well as motility dysregulations such as achalasia, and diffuse esophageal spasms (23–27). The majority of food impactions in EoE patients are thought to be the result of a natural progression from an inflammatory phenotype of EoE to a fibrotic one (28–30). However, particularly in the pediatric population, there are EoE patients who present with food impactions without showing any endoscopic features of fibrosis (27).

Hence, the goal of the current study was to examine a large dataset of mRNA pattern stamps of EoE patients to define a transcriptional signature that could identify EoE patients with food impactions and yield insights about possible pathological causes for this condition in the context of EoE. We further aimed to modify our medical algorithm that calculates a composite probability score for EoE (pEoE) to establish an additional diagnostic score for the identification of EoE patients with food impaction.

## Materials and methods

### Study population

The patients included in this study were enrolled in an observational longitudinal cohort study, performed at Boston Children's Hospital that concentrates on the understanding of the pathophysiology and diagnosis of EoE (17–19, 31, 32). Children between 1 and 18 years of age, who were scheduled for an elective upper gastrointestinal endoscopy at the Division of Gastroenterology at Boston Children's Hospital, due to a clinical suspicion for EoE (such as presenting with dysphagia, regurgitation, feeding intolerance or failure to thrive), were invited to participate. Following written informed consent by the patients and/or their legal guardians, caregivers filled out a questionnaire regarding the subject's medical history, current and past symptomatology, allergic comorbidity, and dietary habits. In addition to clinical biopsies, two study biopsies, one from the proximal and one from the distal esophagus, were obtained during endoscopy from each patient. Additional information on each subject's medical history was obtained by retrospective chart review. All patients were treated independently of this study. Patients were approached to provide follow-up information and additional esophageal biopsies at follow-up hospital visits and endoscopies. Approval for this study was obtained by the institutional Investigational Review Board of Boston Children's Hospital (Harvard Medical School, Boston MA, approval number: 07-11-0460).

### Clinicopathological diagnosis by reference standards

Patients were classified as having EoE according to existent clinicopathological diagnostic guidelines at the time of sample collection, using the gold standard criteria: esophagitis that is histologically characterized by ≥15 eosinophil per high power field in at least one biopsy obtained after ≥4 weeks of treatment with a PPI and exclusion of other causes of esophageal eosinophilia. Patients that have normal esophageal histology with no clinical evidence of an underlying esophageal disease were classified as controls. Gastroesophageal reflux disease was diagnosed when there was evidence of esophagitis (< 15 eosinophils per hpf after >8 weeks PPI treatment) and symptoms associated with reflux. These patients were excluded from analysis along with those with an unknown or ambiguous diagnosis, received steroid therapy or other immunomodulatory medications at the time of inclusion and/or showed evidence of a narrow esophagus or esophageal strictures.

Food impaction in EoE patients (EoE+FI) was defined as following: (1) Experiencing an episode of food impaction that requires removal of the food bolus via endoscopy or surgery, (2) experiencing an episode in which the patient presents to an emergency department and has radiological evidence of impaction and/or relief of symptoms following administration of esophageal relaxants, (3) experiencing an episode of self-reported food impaction that was resolved by bolus regurgitation. EoE patients that do not meet any of these criteria were designated as EoE without food impaction (EoE no FI). Patients who have been diagnosed with esophageal motility disorders such as achalasia, or who have anatomical abnormalities in the esophagus such as esophageal atresias, or tracheoesophageal fistulas were excluded from the study.

### Biopsy processing and digital mRNA profiling

Study biopsies from the esophagus were collected in RNA*later* (Qiagen, Valencia, CA) and stored at −80°C. For mRNA profiling, the biopsies were thawed and homogenized in RTL buffer (Qiagen). Further processing of the samples was done with the nCounter® Prep Station and Digital Analyzer, following the manufacturer's instructions (nCounter® system; NanoString Technologies, Seattle, WA; www.nanostring.com) using a previously published panel as established based on the published EoE transcriptome (33).

### Statistical analysis

Transcript data was normalized by performing background subtraction and normalizing to the geometric mean of the internal positive controls and to the geometric mean of 5 housekeeping genes. The normalized data was analyzed using Kruskal-Wallis test with Dunn's multiple comparisons test. Disease probability scores were calculated using an established algorithm (17). Statistical analysis of clinical characteristics of patients was performed with Fisher's exact test. All statistical tests were performed using python 3.5.2 with the following modules: scipy 0.18.1, numpy 1.11.1, pandas 0.18.1, scikit-learn 0.18, and matplotlib 1.5.3, or IBM SPSS Statistics for Windows, Version 23.0 (Armonk, NY), or GraphPad Prism 7.00 for Windows (La Jolla, CA).

## Results

### Basic characteristics of EoE patients with food impactions

**Figure 1:**
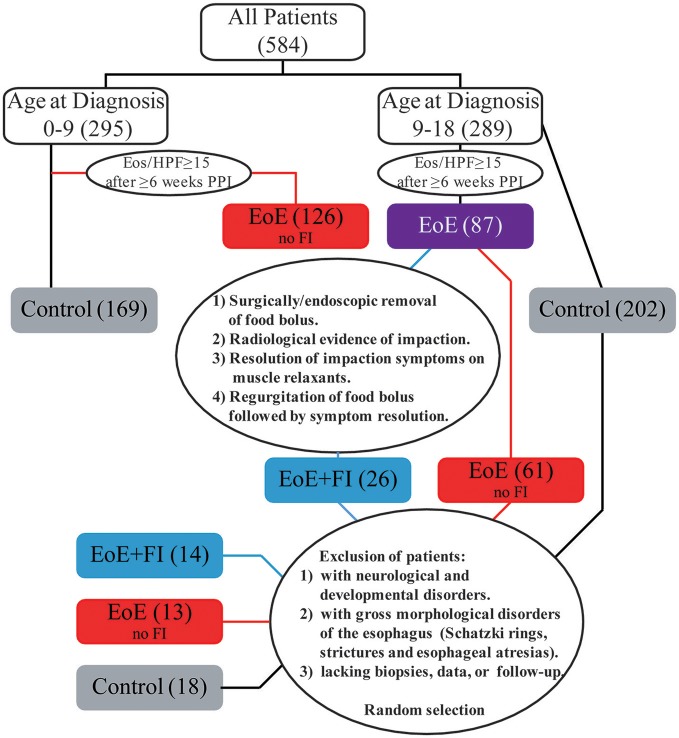
Patient cohort overview.

The incidence of FI in the EoE population was 12.1% (26/215) referred to as EoE+FI for the rest of the manuscript). While the median age of onset in the pediatric EoE cohort was 10.38 (1.23–18.90), the median age of EoE+FI patients was 14.79 (10.70–16.99), implying that FI is found in a subpopulation of patients with onset and/or diagnosis later in childhood. When restricting the analysis to EoE patients that were diagnosed after the age of nine, the incidence of FI increased to 29.2% (26/89). To control for age as a confounding factor for the mRNA pattern comparison, we randomly selected 13 age-matched EoE patients who did not present with a food impaction event prior or at least 2 years after diagnosis and 18 age-matched control patients with no esophageal eosinophilia or esophageal inflammation (Figure 1). This patient population was used for comparative mRNA pattern analysis and machine learning strategies presented in the rest of the manuscript.

EoE is predominantly found in males (34). In our entire cohort, 67.0% (144/215) of patients were male. In a sub-analysis of the age–matched study population an even higher male predominance of cases presenting with FI was observed with 93% (13/14) of EoE+FI being male compared to 62% (8/13) in the age-matched EoE group without FI.

The incidences of common gastrointestinal symptoms such as epigastric pain, reflux symptoms, and vomiting were comparable between EoE patients who did or did not present with FI. In contrast, dysphagia was significantly more common in the EoE+FI group. The gross endoscopic findings, such as esophageal pallor, edemas, furrowing, loss of vascularity, and the presence of exudate, were comparable between EoE patients with and without FI (Table 1).

**Table 1:** Patient characteristics and cohort composition.

**Parameter**	**EoE+FI**	**EoE no FI**	**Controls**	***P*****-values**		
				**EoE-FI vs. EoE no FI**	**EoE-FI vs. Controls**	**EoE no FI vs. Controls**
*n*	14	13	18
Age at diagnosis (in years; median, range)	14.26 (10.70–17.58)	13.41 (9.08–15.72)	13.39 (10.63–17.98)	0.528	>0.999	0.466
Male gender	13/14 (93%)	8/13 (62%)	6/18 (33%)	0.077	< 0.001	0.157
**SYMPTOMS IN THE PAST YEAR**						
Dysphagia	14/14 (100%)	9/13 (69%)	5/18 (28%)	0.041	< 0.001	0.033
Food impaction	14/14 (100%)	0/13 (0%)	0/18 (0%)	NA	NA	>0.999
Chest pain	3/14 (21%)	0/13 (0%)	2/18 (11%)	0.222	0.631	0.497
Epigastric pain	4/14 (29%)	6/13(46%)	9/18 (50%)	0.440	0.289	>0.999
Reflux symptoms	4/14 (29%)	6/13(46%)	11/18 (61%)	0.440	0.087	0.481
Feeding difficulties	0/14 (0%)	0/13 (0%)	0/18 (0%)	1.000	>0.999	>0.999
Vomiting	2/14 (14%)	4/13 (31%)	2/18 (11%)	0.385	>0.999	0.208
**ENDOSCOPY**						
Pallor	3/14 (21%)	1/13 (8%)	1/18 (6%)	0.596	0.295	>0.999
Edema	1/14 (7%)	0/13 (0%)	0/18 (0%)	>0.999	0.438	>0.999
Loss of vascularity	7/14 (50%)	2/13 (15%)	0/18 (0%)	0.103	0.001	0.168
Furrowing	11/14 (79%)	9/13 (69%)	3/18 (17%)	0.678	< 0.001	0.008
Exudate	6/14 (43%)	5/13 (38%)	0/18 (0%)	>0.999	0.003	0.008
**ALLERGIC/ATOPIC CONDITIONS**						
Serum IgE levels (median, range)	214 (63–503)	100.5 (4–1920)	98 (35–189)	0.733	0.519	>0.999
Eczema	5/14 (36%)	3/13 (23%)	1/18 (6%)	0.678	0.064	0.284
Asthma	8/14 (57%)	5/13 (38%)	1/18 (6%)	0.449	0.004	0.059
Allergic rhinoconjunctivitis	7/14 (50%)	9/13 (69%)	4/18 (22%)	0.440	0.142	0.013
Food allergy	4/14 (29%)	3/13 (23%)	0/18 (0%)	>0.999	0.028	0.064
Positive RAST or skin prick test against food antigens	11/14 (78%)	8/13 (61%)	0/5 (0%)	0.420	0.005	0.036
**TISSUE EOSINOPHILIA (PEAK VALUE)**						
Proximal (median, range)	25 (0–110)	25 (0–100)	0	>0.999	< 0.001	< 0.001
Distal (median, range)	50 (3–80)	70 (25–150)	0	>0.999	< 0.001	< 0.001
Maximum eosinophil count (median, range)	89 (0–115)	70 (25–150)	0	>0.999	< 0.001	< 0.001

*p-values calculated by Fisher's exact test*.

### EoE patients with food impactions cannot be differentiated from other EoE patients based on standard diagnostic measures

**Figure 2:**
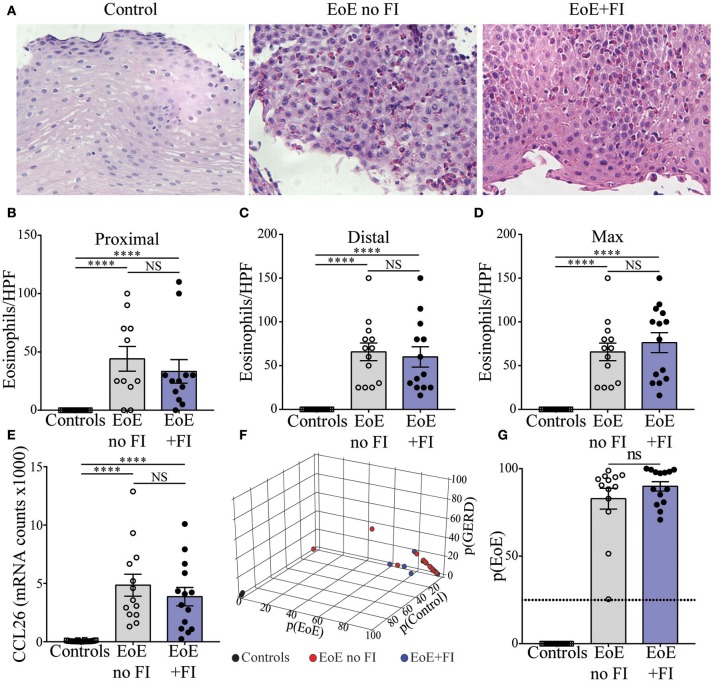
Measures of EoE severity.**(A)** Representative hematoxylin and eosin staining of distal esophageal biopsies of Control, EoE no FI, and EoE+FI patients. Comparison of eosinophil counts in **(B)** proximal and **(C)** distal esophageal biopsies. **(D)** Maximum eosinophil infiltration. **(E)** CCL2 mRNA transcript levels in the esophagus. **(F,G)** disease probability scores (p(EoE), p(Control), p(GERD)) in Control, EoE no FI, and EoE+FI patients. ^****^p < 0.0001 as calculated by Dunn's multiple comparison test after Kruskal-Wallis test.

Histological and transcriptional measures of eosinophil infiltration were analyzed to determine whether common markers of disease severity could be used to distinguish EoE from EoE+FI patients. No differences in the degree of eosinophilia in either proximal or distal biopsies (Figures 2A–C), or in the maximum count throughout the esophagus was noted (Figure 2D) between the two patient groups. Additionally, the esophageal expression of the eosinotrophic chemokine *CCL26* was not significantly different between EoE no FI and EoE+FI patients (Figure 2E). A recently published diagnostic algorithm uses machine learning approaches to calculate a probability score for EoE diagnosis (p(EoE)) based on the transcriptional profile of the esophageal tissue in EoE patients (17). Based on this algorithm, an EoE diagnosis could be made when p(EoE) values are >25. When calculating the three probability values for EoE, GERD and control patients, EoE no FI, and EoE+FI patients cluster together and separate from control patients (Figure 2F). The p(EoE) of patients with and without FI was not significantly different (Figure 2G). This data suggests that the presentation of food impactions in EoE does not result from a quantitative difference in tissue eosinophilia, which indicates that an additional analysis loop is needed to expand the medical algorithm to better define the EoE+FI subpopulation.

### EoE patients with food impaction present with comparable measures of systemic allergy but decreased levels of esophageal allergy based on the IGHE score

**Figure 3:**
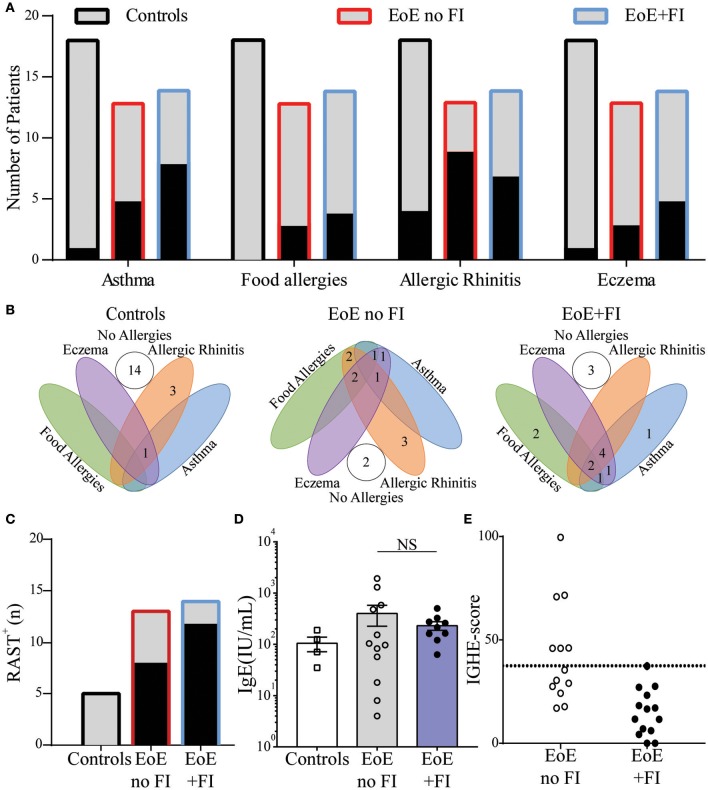
Comparison of clinical allergies and measurements of allergic sensitization in EoE no FI and EoE+FI patients. **(A)** Frequency of individual allergic comorbidities, and **(B)** distribution throughout the patient population. **(C)** Patients with a positive RAST to a food allergen. **(D)** Serum concentrations of IgE. **(E)** Esophageal allergy scores (IGHE score).

Since EoE is classified as an allergic disorder, allergic comorbidities were analyzed as a potential factor in distinguishing the clinically-defined EoE+FI patient subpopulation. No significant difference in the occurrence of food allergies, asthma, eczema, or seasonal allergies was observed in EoE+FI patients compared to EoE patients without FI (Figures 3A,B). The frequency of sensitized patients as determined by RAST or skin prick tests was also comparable between both patient groups (Figure 3C). Furthermore, there was no significant difference in total serum IgE titers (Figure 3D). These data suggest that there is no relationship between IgE mediated allergic comorbidities and food impactions in EoE patients. It is important to note here, however, that the correlation between serum IgE titers and EoE is low (35, 36). We recently established esophageal IgE production as an additional readout for tissue allergy in EoE patients (17). To test if esophageal tissue allergy can be used to identify EoE+FI patients, we analyzed the composite IGHE score. This score was defined as a secondary analysis loop of the published EoE diagnostic algorithm as a correlative measure of increased esophageal allergic Th2-type inflammation (17). Using the published cutoff score of 37.5, we found that 6/13 patients without FI presented with an elevated IGHE-score while none of the patients with FI did (Figure 3E). Based on this analysis, the systemic allergic phenotype is unlikely an eliciting and/or contributing factor for the development of food impaction in EoE patients, however the extent of the local esophageal allergic phenotype may negatively correlate with FI symptoms.

### Esophageal inflammation in EoE patients with food impaction differs from those without food impaction

**Figure 4:**
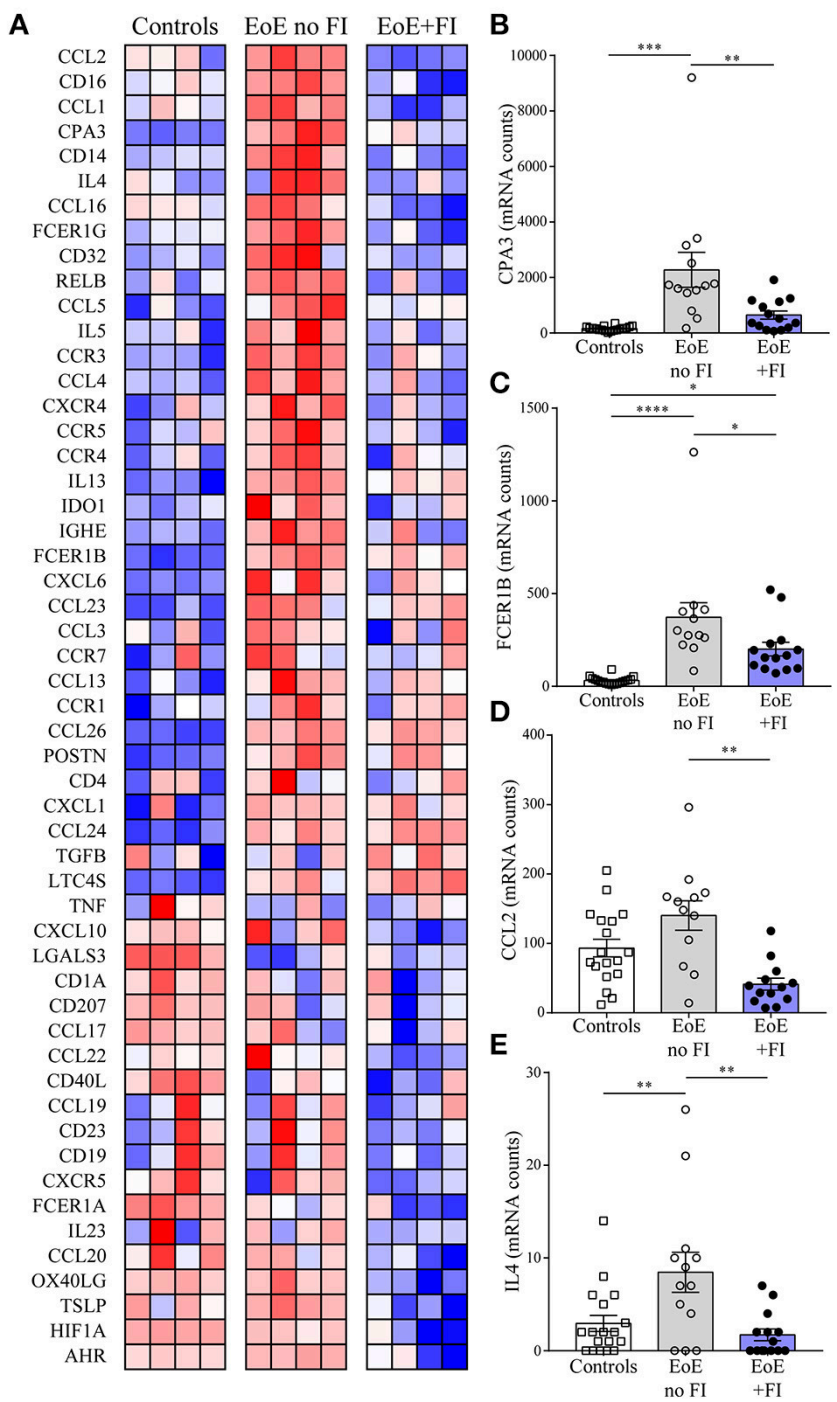
EoE+FI specific mRNA pattern. **(A)** Heat map comparison of esophageal mRNA patterns in control, EoE no FI, and EoE+FI patients. Relative expression of **(B)** CPA3, **(C)** FCER1B, **(D)** CCL2, **(E)** IL4 in control, EoE no FI, and EoE+FI patients. ^*^p < 0.05 ^**^p < 0.01, ^***^p < 0.001,^****^p < 0.0001 as calculated by Dunn's multiple comparison test after Kruskal-Wallis test.

EoE is a complex multifactorial disease, so in order to understand the immunological pathways contributing to the differences in disease presentation the transcriptional profile of 74 genes was analyzed (Figure 4A). EoE+FI patients present with significantly lower transcript counts of *CPA3*, a mast cell specific transcript that encodes for carboxypeptidase a3, as well as lower *FCER1B*, the mast cell and basophil specific beta chain of the high-affinity IgE receptor (Figures 4B,C). In agreement with the IGHE score, this data set suggests that EoE+FI patients present with a lesser contribution of the esophageal mast cell compartment to the local inflammation. *CCL2* is the primary chemokine responsible for the recruitment of mast cell precursors. In line with the probable lower frequency of mast cells, patients presenting with food impactions express lower transcript levels of *CCL2* in their esophageal biopsies indicating that the recruitment of this cell type might be altered (Figure 4D). Finally, it is important to note that the Th2-type cytokines *IL4* and *IL5* are significantly less expressed in patients with food impaction, suggesting a less pronounced Th2-type inflammatory tissue environment in patients with FI (Figures 4A,E). Combined these data demonstrate that patients with food impaction present with similar levels of tissue eosinophilia but the mast cell infiltration and Th2 inflammation are reduced.

### Establishing an analysis loop that allows for the differentiation of EoE patients with food impactions

**Figure 5:**
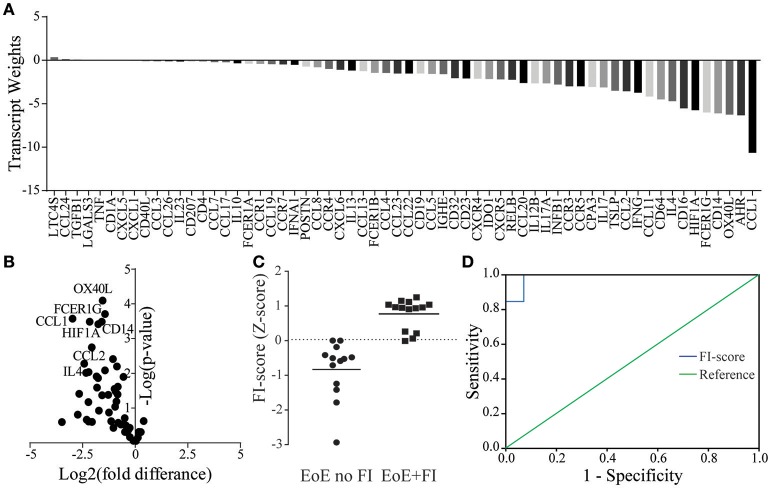
FI-score in EoE no FI and EoE+FI patients**. (A)** Transcript weights of the factors differentiating EoE and EoE+FI. **(B)** Volcano plots of normalized mRNA transcripts displayed as fold difference (x-axis) and significance (y-axis) used for the calculation of the factor weights. **(C)** Calculated standardized FI-score. **(D)** ROC analysis for differentiating EoE no FI and EoE+FI patients based on FI-score (AUC = 0.99, Sensitivity = 0.93 Specificity = 1).

Given the unique characteristics in immunological mRNA transcript pattern of EoE patients with FI, we hypothesized that an algorithm-based score could be developed to differentiate EoE patients with and without FI. By weighing transcripts based on the fold difference in their expression and their adjusted significance, transcript weights were calculated (Figures 5A,B). These weights were used to calculate a raw composite FI-score for each EoE patient using weighted factor analysis. This raw score was then standardized and a cutoff determined by ROC analysis. With the calculated cutoff of 0.03 the FI-score distinguished patients with and without FI with a sensitivity of 0.93 and a specificity of 1.00 (Figures 5C,D). The AUC for the ROC was 0.99 with a standard error of 0.02 and a positive predictive value of 100.00% and a negative predictive value of 92.86%. This secondary analysis can be used to expand the previously published diagnostic algorithm and may serve to predict the risk of food impactions prospectively.

### The inflammatory mRNA pattern of EoE+FI patients indicates underlying esophageal dysmotility

**Figure 6:**
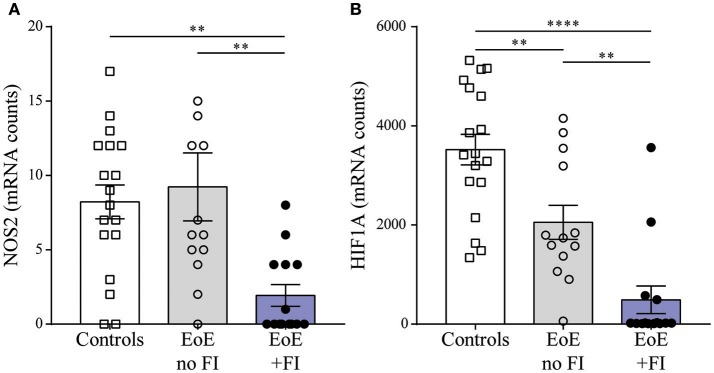
Motility related transcript levels. Comparison of **(A)** NOS2 and **(B)** HIF1A expression between patient groups. ^**^p < 0.01, ^***^p < 0.001 as calculated by Dunn's multiple comparison test after Kruskal-Wallis test.

When analyzing the mRNA pattern for transcripts with potential influence on motility, we found that the inducible nitric oxide (NO) synthase *NOS2* was significantly less expressed in EoE patients with FI (Figure 6A). Therefore these patients may not produce sufficient esophageal NO, a key signal for relaxation of smooth muscles (37, 38). Additionally, the expression levels of the transcription factor hypoxia induced factor 1a (*HIF1A*, Figure 6B) were significantly lower in EoE+FI patients. Loss of *HIF1A* expression has previously been described to result in an increase in smooth muscle contractility (39, 40). Combined, these results show altered expression levels of key regulators of motility, which implies that esophageal dysmotility may underlie the development of FI in EoE.

## Discussion

Our work confirmed that EoE patients who suffer from food impactions cannot be identified as a distinct subgroup of EoE using gold standard diagnostic markers, such as eosinophil infiltration, histological features, or comorbidities. Yet, we were able to identify a unique esophageal mRNA profile in EoE patients with food impactions who did not show any evidence of anatomical narrowing of the esophagus by endoscopy. A modification of a published medical algorithm used the FI-specific mRNA pattern stamp to create a diagnostic score for this patient subpopulation (EoE+FI), which is characterized by lower expression levels of mast cell specific transcripts, Th2 cytokines, and decreased expression of key regulators of smooth muscle contractility and relaxation.

Food impaction is a common complication in EoE. In adult onset EoE, FI occurs with high frequency (55%) and is one of the main indicators of the disease (41). However, in our pediatric cohort, it occurred in only 12% of our cohort, which probably reflects the fact that in children FI start to occur in older children. As in adults we also observed a male predominance (35). Currently, no diagnostic tool for the identification of patients prone to develop this comorbidity exists. Generally, EoE is a chronic inflammatory disorder that leads to fibrosis and strictures in the esophagus and it has been thought that food impactions occur due to the fibrostenotic complications. Indeed strictures are found and identified as the cause of food impaction in some of the patients. However, in many patients, food impactions occur in the absence of such anatomical problems or any other identifiable cause. These observations imply that impaired esophageal motility contributes to food impactions among pediatric EoE patients (27). The hypothesis that food impactions are not necessarily connected to fibrosis is strongly supported by the findings from our study, which indicate that markers of mast cell expansion and Th2 cytokines, both of which are key regulators of fibrosis, are downregulated in EoE patients with food impactions. The observed inflammatory mRNA pattern implies that FI in pediatric EoE patients may develop independent of fibrosis and rather, manifests as a result of esophageal dysmotility.

Transcripts for inducible nitric oxide synthase (*NOS2*) were significantly downregulated in EoE+FI patients. Lower expression of *NOS2* suggests diminished production and lower bioavailability of NO. This may lead to an increase in smooth muscle cell contractility because lack of NO prevents smooth muscle relaxation, which in turn results in poor peristalsis. Additionally, *HIF1A* was downregulated in EoE+FI patients in our cohort. HIF1A expression levels are acknowledged for contributing to the regulation of vascular smooth muscle tone and low levels of *HIF1A* expression have been shown to be associated with hypertension due to hypercontractile vascular smooth musculature (39, 40). It is currently unknown whether the role of *HIF1A* in smooth muscle contractility applies to esophageal smooth muscle cells. However, it is tempting to speculate that the reduction in esophageal *HIF1A* expression levels contributes to motility dysfunction by exacerbating the difficulty of esophageal smooth muscles to relax during swallowing. The combination of low *NOS2* expression to regulate relaxation and low *HIF1A* expression to regulate smooth muscle tone and contractility can potentially result in smooth muscle cramping and spasm, and subsequently to food getting stuck in the esophagus in a fibrosis-independent manner.

Using both the immunological and non-immunological transcriptional differences in the expression patterns of EoE patients with food impactions, we modified our existing algorithm for diagnosing EoE patients to include a secondary analysis for assessing the risk for food impaction. The predictive power of the current study is limited by the small number of pediatric EoE patients with food impactions. It will, therefore, be important to confirm the accuracy of our algorithm by recruiting additional patients who can be integrated into the current analysis in a forward-feeding way to expand the training set and to generate an additional test set. Ideally, such an analysis would be performed as a multicenter study. Another limitation of the current study is its retrospective nature which allows for the identification of a gene signature that identifies patients with food impactions as a subpopulation but cannot predict the development of this complication. For the latter purpose, a longitudinal cohort study needs to be designed to predictively analyze the esophageal mRNA signature changes in EoE patients as they age and as they develop food impaction, or phenotypes specific to other subpopulations of EoE. Such analysis will determine if the differences in gene expression, which we have identified as specific for EoE+FI can be observed before the first food impaction event. Furthermore, at the inception of our cohort, the definition of EoE still included a lack of response to 8 weeks of PPI, so all patients included in the present cohort are EoE patients that did not respond to PPI. We do not have any patient with PPI responsive eosinophilia, in the study cohort so it is not clear if the mRNA pattern of those patients will be the same.

Currently, the unpredictability of EoE therapy response and disease progression necessitates physicians to find the best treatment strategy for each patient using a trial and error approach monitored by gold standard diagnosis which significantly affects the quality of life of the EoE patients (42). For this purpose, EoE algorithms and their future expansions with secondary diagnostic loops appear as attractive strategies because they will permit the prediction of the development of symptoms and treatment responsiveness and can, thus, help inform the diagnosis and treatment of EoE reducing the time between initial presentation and effective treatment. Improved diagnostic strategies will assist physicians in educating EoE patients and their families if their score implies the risk of developing food impactions, thus helping the child and family anticipate how the disease will impact them particularly. Additionally, the FI-score will assist physicians in assessing the risk for food impactions in each patient objectively, making it a highly useful diagnostic tool when dealing with patients that are unable to accurately express their symptoms.

## Author contributions

BS, UA, CK, RR, SB, and EF designed experiments, performed research, and analyzed data. KH, RR, JV, JG, and SN were involved in patient recruitment, evaluation of clinical patient status, evaluation of biopsies, and analysis of human serum data. BS, SN, and EF wrote the first draft of the manuscript. All co-authors contributed to writing the final manuscript and approved its last version.

### Conflict of interest statement

The authors declare that the research was conducted in the absence of any commercial or financial relationships that could be construed as a potential conflict of interest. The handling Editor declared a shared affiliation, though no other collaboration, with the authors and the reviewer HO.

## Abbreviations

FI: Food impaction
AUC: area under the curve
EoE: eosinophilic esophagitis
EoE+FI: eosinophilic esophagitis presenting with food impaction
GERD: gastroesophageal reflux disease
PPI-REE: proton pump responsive eosinophilic esophagitis
NO: nitric oxide
*HIF1A*: hypoxia induced factor 1a
*CCL26*: C-C motif chemokine ligand 26
*NOS2*, Nitric oxide synthase 2, inducible nitric oxide synthase *IL4*: Interleukin 4
*IL5*: Interleukin 5
*FCER1B*, Fc fragment of IgE, high affinity I: receptor for
beta polypeptide: High affinity IgE receptor beta polypeptide
*CPA3*: carboxypeptidase a3.
